# Improved spectral resolution and high reliability of *in vivo* ^1^H MRS at 7 T allow the characterization of the effect of acute exercise on carnosine in skeletal muscle

**DOI:** 10.1002/nbm.3447

**Published:** 2015-11-29

**Authors:** Ivica Just Kukurová, Ladislav Valkovič, Jozef Ukropec, Barbora de Courten, Marek Chmelík, Barbara Ukropcová, Siegfried Trattnig, Martin Krššák

**Affiliations:** ^1^ High Field MR Centre, Department of Biomedical Imaging and Image‐guided TherapyMedical University of ViennaViennaAustria; ^2^ Christian Doppler Laboratory for Clinical Molecular MR ImagingViennaAustria; ^3^ Department of Imaging MethodsInstitute of Measurement Science, Slovak Academy of SciencesBratislavaSlovakia; ^4^ Oxford Centre for Clinical Magnetic Resonance Research (OCMR)University of OxfordOxfordUK; ^5^ Obesity Section, Diabetes and Metabolic Disease LaboratoryInstitute of Experimental Endocrinology, Slovak Academy of SciencesBratislavaSlovakia; ^6^ Monash Centre for Health, Research and ImplementationSchool of Public Health and Preventive MedicineMelbourneAustralia; ^7^ School of MedicineCommenius University BratislavaBratislavaSlovakia; ^8^ Division of Endocrinology and Metabolism, Department of Internal Medicine IIIMedical University of ViennaViennaAustria

**Keywords:** carnosine, ^1^H MRS, 7 T, skeletal muscle, acute exercise, relaxation times, repeatability

## Abstract

The aims of this study were to observe the behavior of carnosine peaks in human soleus (SOL) and gastrocnemius (GM) muscles following acute exercise, to determine the relaxation times and to assess the repeatability of carnosine quantification by ^1^H MRS at 7 T. Relaxation constants in GM and SOL were measured by a stimulated echo acquisition mode (STEAM) localization sequence. For *T*_1_ measurement, an inversion recovery sequence was used. The repeatability of the measurement and the absolute quantification of carnosine were determined in both muscles in five healthy volunteers. For absolute quantification, an internal water reference signal was used. The effect of acute exercise on carnosine levels and resonance lines was tested in eight recreational runners/cyclists. The defined carnosine measurement protocol was applied three times – before and twice after (approximately 20 and 40 min) a 1‐h submaximal street run and additional toe‐hopping. The measured *T*_1_ relaxation times for the C2‐H carnosine peak at 7 T were 2002 ± 94 and 1997 ± 259 ms for GM and SOL, respectively, and the *T*_2_ times were 95.8 ± 9.4 and 81.0 ± 21.8 ms for GM and SOL, respectively. The coefficient of variation of the carnosine quantification measurement was 9.1% for GM and 6.3% for SOL, showing high repeatability, and the intraclass correlation coefficients (ICCs) of 0.93 for GM and 0.98 for SOL indicate the high reliability of the measurement. Acute exercise did not change the concentration of carnosine in the muscle, but affected the shape of the resonance lines, in terms of the shifting and splitting into doublets. Carnosine measurement by ^1^H MRS at 7 T in skeletal muscle exhibits high repeatability and reliability. The observed effects of acute exercise were more prominent in GM, probably as a result of the larger portion of glycolytic fibers in this muscle and the more pronounced exercise‐induced change in pH. Our results support the application of the MRS‐based assessment of carnosine for pH measurement in muscle compartments. © 2015 The Authors. NMR in Biomedicine published by John Wiley & Sons Ltd.

**Abbreviations used**
ANOVA: analysis of variance
BMI: body mass index
CV: coefficient of variation
GM: gastrocnemius muscle
HSVLD: Hankel Lanczos Squares Singular Values Decomposition
ICC: intraclass correlation coefficient
RF: radiofrequency
SNR: signal‐to‐noise ratio
SOL: soleus muscle
STEAM: stimulated echo acquisition mode.

## Introduction

Carnosine (β‐alanyl‐l‐histidine) is a cytoplasmic dipeptide present in the human body, mainly in skeletal muscle. Its known physiological functions include pH buffering, anti‐inflammatory and anti‐oxidative properties, and protection against the formation of advanced glycation and lipoxidation end‐products 1, 2. Its complete and definitive role in muscle physiology is still not fully understood.

The concentration of carnosine in the muscle varies inter‐individually in the range of 5–8 mmol/kg wet weight (or 20–30 mmol/kg dry weight) 2, 3, 4. The levels of carnosine in muscle are relatively stable 5 as carnosinase is not present in muscle 6. There are multiple factors that probably influence muscle carnosine content. The main determinants are muscle fiber type composition (glycolytic/oxidative), age, sex, muscle training state and nutritional factors, including oral supplementation with the carnosine precursor amino acid β‐alanine 2, 7, 8, 9, 10, 11, 12. To date, no evidence of the upper limit for muscle carnosine content has been described. Moreover, oral supplementation has been shown to increase carnosine levels by more than 80%, with relatively slow recovery to pre‐intervention levels 4, 5, 13, 14. Although its contribution to overall buffering capacity is only expected to be around 10–20% 15, 16, its concentration determinants make this skeletal muscle pH‐buffering metabolite the only one that can be manipulated externally.

Carnosine can be non‐invasively detected by ^1^H MRS. Two spectral lines arising from C2‐H and C4‐H protons on the imidazole ring resonate downfield from water at approximately 8 and 7 ppm, respectively. The chemical shift of these lines is known to be pH sensitive. This was shown *in vivo* 3, 17, in good agreement with concurrent pH measurement by ^31^P MRS. However, orientation‐dependent effects, based on dipolar coupling, chemical shift anisotropy and possible *T*_2_ anisotropy 18, 19, 20, 21, as in other skeletal muscle metabolites, i.e. creatine, taurine and lactate, must be taken into account when evaluating carnosine ^1^H MRS data. Broadening of the resonance lines, splitting and the appearance of satellites are observed when orientation of the muscle fibers to the magnetic field is further away from the magic angle. This effect is more pronounced at the C4‐H peak at 7 ppm. Therefore, the C2‐H peak at 8 ppm, with the additional advantage of a longer *T*_2_, is more suitable for analysis 3, 18, 21.

To date, ^1^H MRS has been used to assess the concentration of carnosine in *in vivo* human spectroscopic studies or to measure pH by ^1^H spectroscopy at magnetic fields of 1.5, 3 or 4.7 T 3, 22, 23. Apart from studies focusing on training and its relation to carnosine concentration 5, 9, 24, only a few investigations have focused on carnosine during and shortly after acute exercise, and therefore little is known about its exercise‐related metabolism. Immediately after exhaustive exercise of the human forearm, Pan *et al*. 3 observed a shift of the carnosine resonance ascribed to changes in pH. This observation was confirmed also by interleaved ^31^P MRS and broadening of the C2‐H peak in the post‐exercise spectra. After closer analysis, some splitting of the peak could be noticed, although this was not commented upon in the original work. Later, the changes in the carnosine resonance appearance were ascribed to non‐ideal repositioning or movement of the muscle during exercise, as the carnosine peak is prone to orientation‐dependent effects 18. Nevertheless, in a study on stimulated dissected frog muscles at 7 T, Damon *et al*. 17 showed similar changes in the post‐exercise spectral behavior of the C2‐H peak, particularly its broadening, position shift and obvious splitting of the peak. These effects were interpreted as pH differences related to carnosine presence in two muscular compartments, namely in glycolytic and oxidative muscle fibers. No changes in concentration were observed in any of these studies.

The strong magnetic field of 7 T provides, in general, better sensitivity, better spectral resolution and higher repeatability in comparison to lower magnetic fields. To date, the effects of acute exercise on the behavior of human calf carnosine concentration have not been studied with ^1^H MRS at 7 T. The aims of this study were to describe the behavior of the carnosine peaks at 7 T following a 1‐h street run and to confirm the previous suggestions 3 from lower field studies, which were later described *ex vivo* 17. The focus was the observation of the chemical shifts and changes in the shape of the C2‐H carnosine peak in the soleus (SOL) and gastrocnemius (GM) calf muscles, and on the determination of the concentration of this metabolite, possibly affected by exercise. As a prerequisite for absolute quantification, we aimed to determine the relaxation times of carnosine in SOL and GM, and to assess the repeatability of the measurements by ^1^H MRS at 7 T.

## Materials and Methods

All measurements were performed on a 7‐T whole‐body MR system (Magnetom, Siemens Healthcare, Erlangen, Germany) equipped with a TX/RX 1/28‐channel ^1^H knee radiofrequency (RF) coil (QED, Mayfield Village, OH, USA). The local ethics committee approved the protocol, and written informed consent was obtained from all volunteers. All volunteers were examined in the supine position, with the widest part of the right calf placed in the middle of the RF coil in the magnet isocenter.

### Relaxation times of carnosine

**Figure 1:**
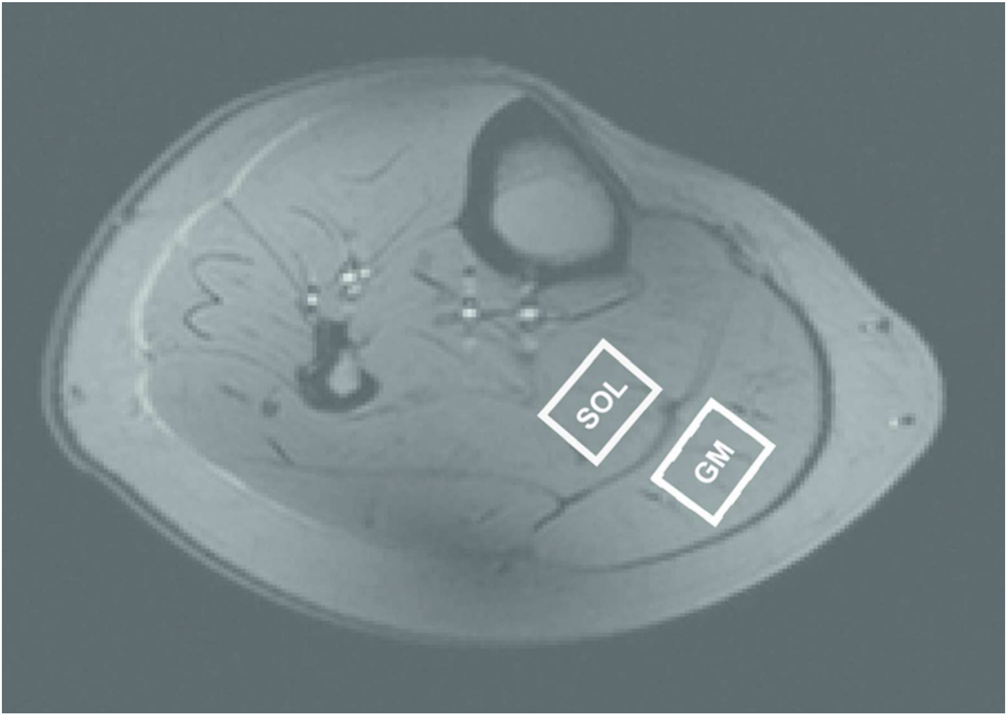
Localizer image with depicted positions of the volumes of interest in the gastrocnemius medialis muscle (GM) and medial part of the soleus muscle (SOL).

Seven healthy volunteers [four women, three men; age, 28.6 ± 4.0 years; body mass index (BMI), 21.02 ± 1.92 kg/m^2^; two female vegetarians] were recruited for the measurement of the relaxation times of carnosine. For localization, *T*_1_‐weighted images were acquired. Shimming was performed on the whole volume of the calf, based on gradient‐recalled, double‐echo field map acquisition (GRE‐SHIM, WIP_452, Siemens Healthcare). Spatial selection was achieved using a stimulated echo acquisition mode (STEAM) localization sequence. The volume of interest was carefully placed in GM or SOL (Fig. 1) with the size ranging from 4 to 7.5 cm^3^, depending on the individual muscle size. To achieve appropriate excitation of the carnosine signals, the excitation frequency was set to 7.5 ppm (between the C2‐H and C4‐H carnosine peaks). The *T*_1_ relaxation time was measured by an inversion recovery sequence with the following parameters: TR = 10 s; TM = 20 ms; TE = 30 ms; TI = 50, 100, 300, 1000, 1500, 3000, 5000, 8000 ms; 20 averages; water suppression with a bandwidth of 80 Hz. For the measurement of the *T*_2_ relaxation time, a STEAM sequence was used with the following parameters: TR = 6 s; TM = 20 ms; TE = 20, 30, 50, 70, 90, 120, 150, 300 ms; 20 averages; two dummy scans; same water suppression as applied for the *T*_1_ measurement. In addition, the values of the *T*_1_ and *T*_2_ relaxation times of water in GM and SOL were measured in four volunteers with the same protocol, but without water suppression, four averages and the excitation pulse centered at 4.7 ppm. The total scan time needed for water and carnosine relaxation time measurements was approximately 70 min.

All spectra were processed in jMRUI software. Residual water and lipid peaks were removed by an Hankel Lanczos Squares Singular Values Decomposition (HSVLD) algorithm 25 from the carnosine spectra, and the C2‐H and C4‐H peaks of carnosine were fitted with AMARES 26 using single Lorentzian line shapes, with linewidths determined empirically and constrained to ±1 Hz.

Relaxation times were calculated in MATLAB (MathWorks, Natick, MA, USA) by fitting the data to the exponential functions: *S*_1_ = *M*_0_ e^–TE/*T*2^ + *c* for *T*_2_ and *S*_1_ = *M*_0_(1 – 2e^–TI/*T*1^) + *c* for *T*_1_.

### Absolute quantification and repeatability

Five healthy volunteers (two women, three men; age, 28.0 ± 3.5 years; BMI, 21.4 ± 2.1 kg/m^2^) underwent test–retest measurement of carnosine concentration by single‐voxel ^1^H MRS. The STEAM sequence was applied with the following parameters: TR = 9 s; TE = 20 ms; TM = 20 ms; 32 measurements; excitation pulse centered at 7.5 ppm; water suppression with a bandwidth of 80 Hz. In addition, spectra without water suppression with the pulse centered at 4.7 ppm were acquired with the same parameters, but only four measurements. The retest measurements were performed after a short break during which the volunteers stayed in the scanner room, and full, careful repositioning and shimming procedures were performed with the same measurement protocol The entire repeatability protocol, including repositioning and re‐shimming, took up to 1 h.

Individual transients were phased, frequency corrected if necessary and summed in jMRUI software. Residual water and lipid peaks were removed by an HSVLD algorithm from the carnosine spectra. As a result of the more stable behavior (less orientation‐dependent features), longer *T*_2_ relaxation and higher signal‐to‐noise ratio (SNR) 3, 9, 19, 22, only the C2‐H peaks of carnosine and water were analyzed further. They were fitted in AMARES using single Lorentzian line shapes without constraints. Values were corrected for transverse and longitudinal relaxation using relaxation constants determined in a previous step.

The absolute concentration of carnosine was calculated according to the formula for molar concentration in wet weight:

(1) $$C_{cn}\;=\;\left(S_{cn}/S_{w}\right)\;\times \;\left({CF}_{w}/{CF}_{cn}\right)\;\times \;c_{w}n_{w}w_{\%}$$

where *S* are the signals of metabolites (cn, carnosine; w, water), CF are the correction factors for relaxation, *c*_w_ = 55 mol/L is the molar concentration of water, *n*_w_ = 2 is the number of protons in a water molecule and *w*_%_ is the approximate water content of skeletal muscle tissue, i.e. 0.7 L/kg wet weight of tissue.

To assess the repeatability of carnosine concentration determination, the mean coefficient of variation (CV) was calculated for test–retest measurements for individual muscles. The reliability of the measurement was analyzed using SPSS (version 21.0; IBM SPSS, Chicago, IL, USA) to compute the intraclass correlation coefficient (ICC) employing a two‐factor mixed‐effects model and type consistency 27, 28.

### Effect of acute exhaustive exercise on skeletal muscle carnosine concentration

**Figure 2:**
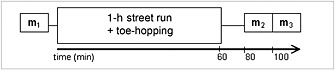
Design of the experiment with three ^1^H MRS measurements (m_1_, m_2_ and m_3_) and their respective timing.

For the application of the method, eight healthy volunteers (recreational runners and cyclists; seven men and one woman; age, 34.5 ± 4.2 years; BMI, 22.8 ± 1.4 kg/m^2^) underwent a study consisting of the measurement of the carnosine concentration before and immediately after a 1‐h submaximal street run. Each measurement was performed with the same parameters as in the repeatability study. After the baseline measurement (m_1_), volunteers went for a run in the vicinity of the MR facility. The average distance covered was 11.4 ± 0.4 km. In addition to the 1‐h run, the volunteers performed toe‐hopping (~100 times) to further stimulate their GM muscle immediately before they were positioned in the magnet for the second measurement. The delay between the end of the run and the acquisition of the spectra (m_2_) in GM was approximately 20 min and in SOL approximately 30 min. This was again followed by another cycle of measurements in both muscles (m_3_), with an additional approximately 20‐min delay (Fig. 2).

The spectra were analyzed in a similar manner to the repeatability part of the study. In SOL, the data from all eight volunteers were used. For GM, only seven sets of data were used as a result of a spectral artifact in the baseline spectra. As there were changes in the shapes of the C2‐H carnosine peak detected in the measurements after the run (m_2_), the carnosine peak was fitted with two Lorentzians with no further constraints. The chemical shifts of the peak were converted to pH values, based on the Henderson–Hasselbalch equation and coefficients acquired by Damon *et al*. 17 for a temperature of 37 °C:

$$pH\;=\;6.81\;+\;\log\left[\left(8.57\;–\;\delta \right)/\left(\delta \;–7.65\right)\right]$$

where δ is the chemical shift of the carnosine peak.

For the exact chemical shift of carnosine, spectra were frequency aligned and referenced to the residual water peak at 4.7 ppm. The absolute concentration of carnosine was calculated according to Equation (1). Differences in the values of the carnosine concentration, chemical shift, pH and linewidth of the C2‐H peak between muscle groups at baseline, as well as within each muscle group across the different time points, were tested for significant differences by repeated‐measures analysis of variance (ANOVA) and Bonferroni‐corrected *post hoc* tests in SPSS.

To obtain information about the positioning of the muscle, fiber orientations for GM and SOL for all three measurements were calculated on the basis of creatine peak splitting at 3.9 ppm 20, 21, 29. Mean changes in values between the first two measurements for both muscles and in the mean values for all three measurements in both muscles were calculated in Excel, MS Office.

## Results

### Relaxation times of carnosine

**Figure 3:**
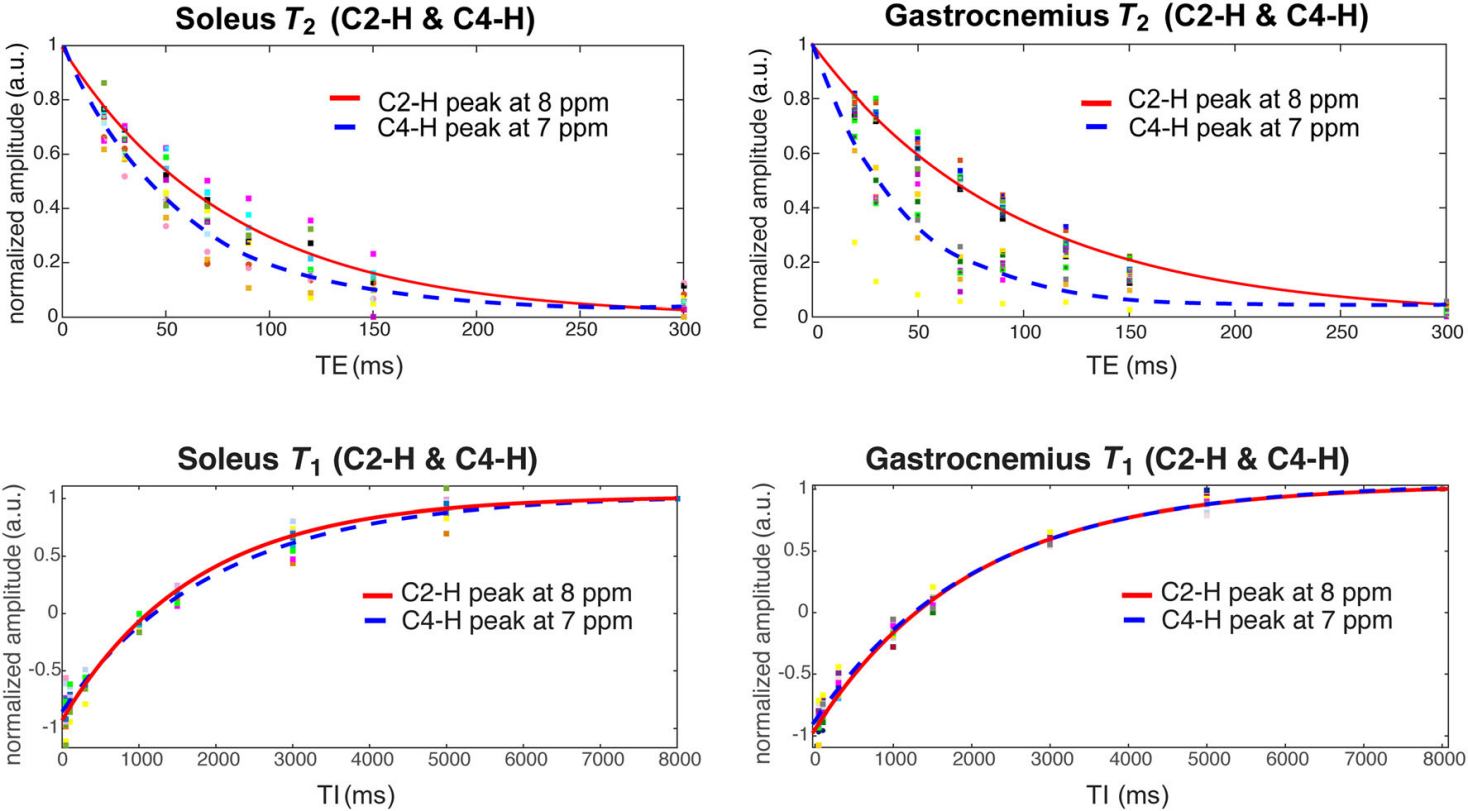
Average fits of *T*_1_ and *T*_2_ relaxation time constants for C2‐H and C4‐H peaks of carnosine in the gastrocnemius medialis and soleus muscles, with individual points in time, all normalized to average values.

Data from GM of all volunteers provided sufficient SNR to fit peaks in the whole range of TE and TI used. In SOL, data from two volunteers did not allow the fitting of the *T*_1_ relaxation time values of the carnosine peaks nor of the *T*_2_ relaxation time values for one volunteer. The results are given in Table 1 and the fitting of the *T*_1_ and *T*_2_ values is depicted in Fig. 3.

**Table 1:** Relaxation time values for C2‐H and C4‐H carnosine peaks and water in milliseconds given with ±standard deviation (SD)

	C2‐H		C4‐H		Water^c^	
	*T*_1_ (ms)	*T*_2_ (ms)	*T*_1_ (ms)	*T*_2_ (ms)	*T*_1_ (ms)	*T*_2_ (ms)
GM^a^	2002 ± 94	95.8 ± 9.4	2039 ± 113	34.1 ± 10.1	1844 ± 83	22.6 ± 1.2
SOL^b^	1997 ± 259	81.0 ± 21.8	1806 ± 289	48.7 ± 16.2	1861 ± 167	23.6 ± 2.3

GM, gastrocnemius medialis; SOL, soleus.

a *n* = 7 for all times of carnosine in GM.

b *n* = 6 for *T*_1_ of carnosine and *n* = 5 for *T*_2_ of carnosine in SOL.

c *n* = 4 for water measurements in both muscles.

### Quantification of carnosine concentration and its repeatability

**Figure 4:**
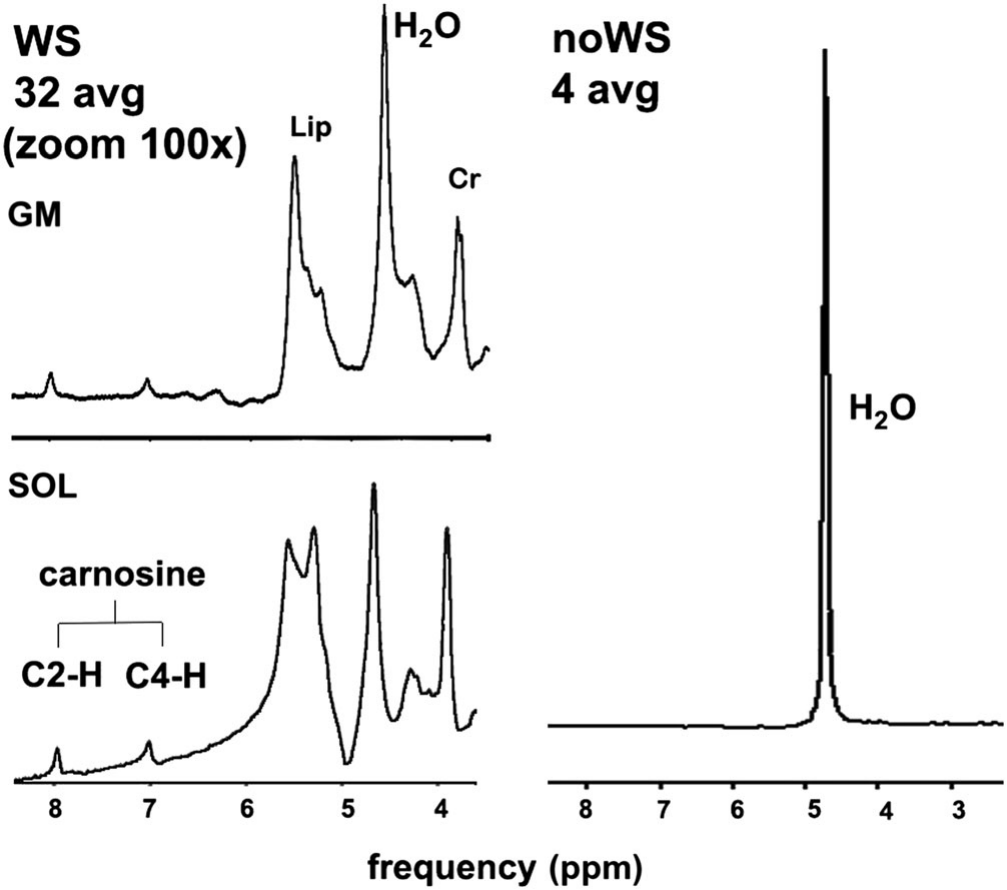
Carnosine spectra from the gastrocnemius medialis (GM) and soleus (SOL) muscles with residual suppressed water signal (WS) and spectrum without water suppression (noWS). Cr, creatine; Lip, lipid.

The carnosine concentration could be quantified in all five volunteers. Examples of the ^1^H MR spectra are shown in Fig. 4. The average concentration of carnosine was 7.15 ± 1.72 mmol/kg wet weight for GM and 6.09 ± 1.88 mmol/kg wet weight for SOL. The mean coefficients of variation were 9.1% and 6.3% for GM and SOL, respectively. Detailed information is given in Table 2. The ICCs were 0.925 for GM and 0.980 for SOL.

**Table 2:** Carnosine concentrations in individual repeated measurements of five volunteers and their means with standard deviation (SD). Values are in mmol/kg wet weight. Individual and mean coefficients of variation (CV) are also given

Volunteer	Sex	GM		CV (%)	SOL		CV (%)
		Test	Retest		Test	Retest	
1	Female	4.80	5.81	13.50	4.52	4.73	3.19
2	Female	6.80	6.03	8.43	4.93	4.56	5.56
3	Male	10.00	9.11	6.76	9.37	9.06	2.36
4	Male	6.78	5.65	12.90	6.15	4.87	16.50
5	Male	8.00	8.44	3.79	6.53	6.19	3.84
Average (±SD)		7.28 (±1.92)	7.01 (±1.63)	9.08	6.30 (±1.91)	5.88 (±1.89)	6.30

GM, gastrocnemius medialis; SOL, soleus.

### Effect of acute exhaustive exercise

The mean concentrations of carnosine detected in GM and SOL before and after exercise are listed in Table 3. ANOVA did not find any significant differences in concentration values.

**Table 3:** Concentrations of carnosine in the gastrocnemius (GM) and soleus (SOL) muscles before and after a 1‐h run. Values are given as mean ± standard deviation (SD)

Time point	GM, *n* = 7, mean ± SD	SOL, *n* = 8, mean ± SD
	(mmol/kg wet weight)	(mmol/kg wet weight)
m_1_	7.8 ± 1.7	5.2 ± 1.4
m_2_	8.7 ± 2.3	6.1 ± 1.4
m_3_	8.4 ± 2.5	5.4 ± 1.6

**Figure 5:**
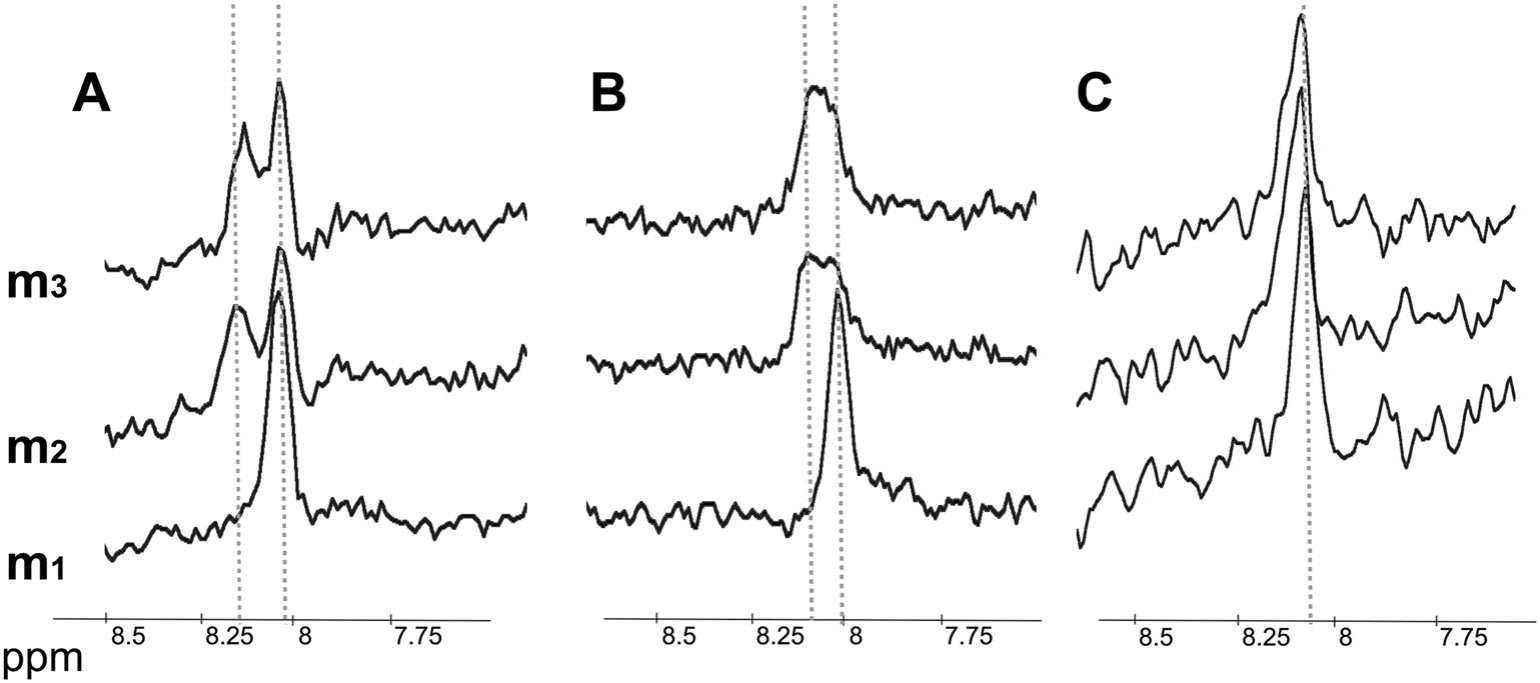
Examples of the C2‐H carnosine peak appearance at 8 ppm following exhaustive exercise at three different time points (m_1_, m_2_ and m_3_) in the gastrocnemius medialis (GM) muscle, with splitting of the peak (A), or widening, and a slight indication of a double peak (B) after exercise. In the soleus (SOL) muscle, there was a remaining single peak without a shift (C). Broken vertical lines depict and reference the positions of the detected resonance lines before and after exercise.

However, exhaustive exercise did cause changes in the shape of the C2‐H peak of carnosine at 8 ppm, more prominently in GM than in SOL. The resonance line in GM became broader and shifted in four volunteers, and was even split into two peaks in three volunteers. To distinguish between these two peaks, the more acidic peak was named GM2 and the peak remaining closer to the original chemical shift position was called GM1. Representative changes in the shapes of the C2‐H peak are depicted in Fig. 5. Average changes in the resonance line parameters for SOL and GM are given in Table 4.

**Table 4:** Characteristics of the carnosine C2‐H peaks before (m_1_) and after (m_2_ and m_3_) exercise

		Chemical shift (ppm)	Acidity (pH)	Linewidth
SOL	m_1_	8.01 ± 0.02	7.01 ± 0.03	12.8 ± 2.4
*n* = 8	m_2_	8.00 ± 0.02	7.00 ± 0.03	15.4 ± 2.3
	m_3_	8.02 ± 0.01	7.00 ± 0.03	15.8 ± 4.9
GM1	m_1_	7.99 ± 0.01	7.05 ± 0.02	9.1 ± 2.8
*n* = 7	m_2_	8.01 ± 0.02	7.00 ± 0.04	13.7 ± 3.2
	m_3_	8.02 ± 0.02†	6.99 ± 0.02†	14.1 ± 6.2
GM2	m_1_	7.98 ± 0.01	7.05 ± 0.02	9.8 ± 2.3
*n* = 7	m_2_	8.07 ± 0.03*	6.89 ± 0.05*	13.9 ± 3.4
	m_3_	8.07 ± 0.02†	6.90 ± 0.04†	13.6 ± 1.0

GM, gastrocnemius medialis; SOL, soleus.

Significant differences [analysis of variance (ANOVA) and *post hoc* Bonferroni‐corrected pairwise comparisons] between the time points are depicted as follows:

* for m_1_ *versus* m_2_, *p* < 0.01;

† for m_1_ *versus* m_3_, *p* < 0.01.

An analysis of the changes in shape and chemical shift of the peaks, which were already visible in individual measurements, showed significant differences for GM1 and GM2, but not for SOL. In particular, significant changes were found in the chemical shift and pH between the m_1_ baseline measurement and the m_2_ measurement performed immediately after exercise and between the m_1_ and the later measurement m_3_ (see Table 4).

**Figure 6:**
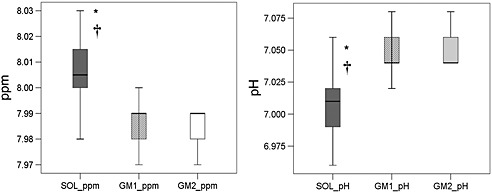
Boxplots of resonance positions (ppm) of the C2‐H carnosine peak and pH value of the respective tissues from baseline measurements in the soleus (SOL) and gastrocnemius medialis (GM: GM1, peak remaining closer to the original chemical shift position; GM2, more acidic peak) muscles. *Significant difference at the *p* < 0.05 level between SOL and GM1 baseline values. †Significant difference at the *p* < 0.05 level between SOL and GM2 baseline values.

When comparing the baseline values of ppm and pH for individual muscles tested by ANOVA, SOL differed from GM1 and GM2 in both parameters (see Fig. 6). In SOL, the pH was slightly more acidic, with an average value of pH 7.01, whereas, in GM, the average value was pH 7.05, which applied to both GM1 and GM2, as they appeared, in most cases, as a single peak at baseline.

An analysis of the fiber orientation during individual measurements is given in Table 5, together with the mean changes in this angle between the first and second measurements after full repositioning.

**Table 5:** Average fiber orientations of the muscles with respect to the magnetic field in individual measurements, and the average change between measurements. Values are given in degrees with standard deviation (SD) (*n* = 8)

		Fiber orientation (deg)	Difference between m_1_ and m_2_
SOL	m_1_	48.0 ± 12.7	
	m_2_	49.0 ± 11.4	2.9 ± 5.6
	m_3_	49.8 ± 9.5	
GM	m_1_	10.0 ± 9.5	
	m_2_	6.4 ± 10.0	6.3 ± 6.8
	m_3_	7.6 ± 13.0	

GM, gastrocnemius medialis; SOL, soleus.

## Discussion

This study shows that ^1^H MRS measurement of carnosine exhibits high repeatability at 7 T, and describes the relaxation times of carnosine in GM and SOL. Although no concentration changes were detected as a consequence of acute exercise, changes in the behavior of the resonance lines were observed.

### Relaxation times of carnosine

The values of the relaxation times assessed here show a pattern similar to that found in previous reports at lower magnetic fields 3, 11, 22, in particular, a shorter *T*_2_ value for the C4‐H peak than for the C2‐H peak, and similar values for GM and SOL.

These are the first *in vivo* assessed values for carnosine in GM muscle at 7 T. Ramadan *et al*. 30 reported relaxation constants of all skeletal muscle metabolites, including carnosine, in SOL. Their *T*_2_ time constants were very similar to ours, but their *T*_1_ values were shorter, most likely because of the different methods of measurement, i.e. progressive saturation used by Ramadan *et al*. *versus* inversion recovery used in this study.

The fact that it was not possible to fit all the spectra from all volunteers in SOL can be explained by the low carnosine SNR in the spectra of two female vegetarian volunteers from our study group. Both factors, sex and vegetarian diet, can contribute to a lower carnosine concentration 11, 12, 15, 23. In addition, because of the different abundance of muscle fiber types, the carnosine content in SOL is known to be lower than that in GM 2, 9, 31.

### Repeatability

The absolute concentration of carnosine was within the range of previously published values – 5–8 mmol/kg wet weight 2. In both muscles, the average concentration for carnosine in women was lower than that in men (4.63 ± 0.08 and 5.86 ± 0.79 for SOL and GM in women *versus* 7.03 ± 1.94 and 8.00 ± 1.68 mmol/kg wet weight for SOL and GM in men), in agreement with previously published results for this adult age group 12. Measurements proved to have high repeatability with CVs of 9.1% for GM and 6.3% for SOL. This is similar to the values already reported at 3 T 5, 22. In all cases, SOL exhibited lower CV for repeated measurements. Although SOL provided less SNR than GM for carnosine, because of the more homogeneous fiber composition in SOL 31 and the less pronounced dipolar effects as a result of the fiber orientation closer to the magic angle, repeatability was better in this muscle. ICC values above 0.9 indicate high reliability of the measurement.

### Effect of exercise

Acute exercise in the form of a 1‐h street run, with additional toe‐hopping, appeared to have no effect on carnosine concentration in either SOL or GM. A possible trend towards an increased concentration of carnosine after exercise was proven to be insignificant (*p* = 0.501). This is in agreement with previous studies, focused on carnosine in different species 3, 17, 32, and confirms the relatively stable concentration of carnosine in muscle. However, we observed alterations in the shape of the C2‐H peak immediately following exercise. This effect was more pronounced in GM, as already observed by Damon *et al*. 17, who studied carnosine with MRS in electrically stimulated dissected frog muscles at 7 T. This was also visible, to some extent, in the spectra from human forearm muscle presented by Pan *et al*. 3 at 4.7 T. Changes in shape include broadening of the peak, a shift to higher ppm values, as well as the appearance of a new peak in the left vicinity of the C2‐H peak. The new peak appearing after exercise was already assigned by Damon *et al*. 17 to the carnosine signal originating in a compartment with a different pH. The changes in chemical shift of the peak observed in our study were not as prominent as in the study by Damon *et al*. 17 (shifts in frog muscle by 0.3–0.5 ppm for the C2‐H peak of carnosine, indicating pH values of 6.2 and 6.8); however, as our exercise paradigm was a submaximal run, and an aerobic endurance activity in recreational runners/cyclists mostly involving oxidative metabolism, it was unlikely to generate an extreme change in intramuscular pH. Similar attempts to study the spectral behavior of the C2‐H peak in the tibialis anterior muscle, after exhausting isometric exercise in humans, with an in‐magnet dorsiflexor ergometer within a 3‐T MR system, also showed a broadening and upfield shift of the resonance line (W. Derave, Ghent University, Belgium, personal communication), but the spectral resolution at lower field strength and/or experimental set‐up were insufficient to deliver the sensitivity for sound conclusions.

We can also consider the appearance of the second line as a splitting of the carnosine peak that mirrors the existence of two skeletal muscle compartments with different pH, possibly as a result of oxidative (slow‐twitch) and glycolytic (fast‐twitch) fiber composition. The pH in slow‐twitch fibers, dependent mainly on oxidative metabolism, changes less than the pH in glycolytic fast‐twitch fibers. The differences in the spectral appearance of the carnosine peaks in GM and SOL after exercise support this hypothesis. SOL has a higher percentage of oxidative fibers (approximately 85%) 31, 33, and spectra from this muscle show almost no exercise‐induced shift and also no splitting of the C2‐H peak (Fig. 5). However, GM, with clearly visible splitting, has only approximately 60% oxidative fibers. Another factor contributing to this difference could be pH regulation, which was proven to be more effective in slow‐twitch fibers 33, 34; thus, no splitting in the SOL spectra and a smaller shift of the GM1 peak could be caused by the faster pH recovery. The spatially heterogeneous recruitment of muscle parts within the same muscle group could also lead to the compartmentalization of acidification. However, the small voxel (4–7.5 cm^3^) used in this study renders the possibility of detecting two differently active muscle compartments within the volume of interest unlikely.

It has been suggested that broadening of the peak can be caused by orientation‐dependent effects as a result of non‐perfect repositioning after exercise 18. In our data, there were no significant changes in the linewidth of the peaks during three consecutive measurements. When analyzed for fiber orientation, the values were similar to those reported previously 20. Changes in GM as a result of repositioning were small and insignificant (6° ± 7°) and even lower in SOL (3° ± 6°), indicating an optimal repositioning with negligible influence on the broadening of the carnosine peak.

Carnosine peaks in the spectra acquired approximately 40 min after the run (m_3_) showed no statistically significant difference from previous measurements (m_2_) for SOL, but the results for the GM1 and GM2 peaks, which showed a significant difference in chemical shift for the m_1_ measurements, suggest that a longer time period is needed to balance the pH in the glycolytic part of the muscle after the exercise challenge applied in this study. Similarly, in the study by Allsop *et al*. 35, in which a maximal sprint exercise was used and in which the pH was measured in the vastus lateralis muscle, no complete pH recovery was detected, even 30 min following the challenge. Nevertheless, we must admit that the observed slow recovery of pH in the present study was unexpected. Further studies with a longer observation period and/or improved time resolution will be needed to describe the full time course of pH changes after a 1‐h submaximal run and subsequent toe‐hopping. Similarly, future investigations using different exercise protocols and cross‐validation with ^31^P MRS and/or muscle biopsies are justified to fully address this issue.

Some variability between volunteers in carnosine spectral behavior following exercise was observed in our data. Different levels of training and hereditary factors, which result in different ratios of glycolytic and oxidative fiber content and oxidative capacity 12, 36, could explain the differences. To confirm this hypothesis, further complementary examinations, e.g. muscle biopsy, should be performed.

In addition to differences in the behavior of the carnosine peaks in individual muscles after exercise, differences between baseline SOL and GM spectra were observed. The most prominent and statistically significant was the difference in chemical shift and pH. In general, these values, particularly for GM, agreed with the data measured by localized ^31^P MRS 37. Other factors could have contributed to this result. It is possible that GM, as a muscle with a different ratio of muscle fiber types than SOL, can manifest more alkaline pH, as found in the case of the extensor digitorum longus 38, 39.

Referencing of the chemical shift is another point that could have contributed to the different results in the respective muscle groups. For this purpose, we used the residual water signal from the partially water‐suppressed spectra as a result of the lower chemical shift displacement error (up to 5 mm) and no orientation‐dependent effects compared with the creatine resonance at 3.05 ppm used previously 17. This approach should ensure close co‐localization of the carnosine resonance and reference line. At this point, we are aware that the temperature dependence of the water resonance frequency (–0.01 ppm/°C ) 40 could have had an effect on the pH quantification. Nevertheless, the observed, rather large, difference in the chemical shift of carnosine (i.e. 0.09 ppm in GM2), and the splitting of the carnosine peak, could not have been caused by temperature changes alone. It should also be noted that a slight shift in the reference value can cause a relatively large error in the resulting pH because of the steepness of the titration curve for the C2‐H carnosine peak at 8 ppm in the normal physiological pH range 17. Therefore, the absolute values of pH, assessed by this method, should be considered with some caution, and the focus should be on the relative changes before and after exercise, as they are influenced by these possible imperfections in the same way. To investigate the differences between muscles objectively, further measurement with various orientations of the muscles in the magnet, and complementary localized ^31^P MRS in SOL and GM, should be performed.

## Conclusions

The present study shows that ^1^H MRS measurements of carnosine in SOL and GM muscles at 7 T exhibit high repeatability and reliability. The measured relaxation times of the carnosine resonance lines in these two muscles provide the necessary information for carnosine quantification in further spectroscopic studies on this metabolite. No effect of acute exercise, in the form of a submaximal run with toe‐hopping, was observed on the carnosine concentration levels in the calf muscles. However, changes in the shape of the resonance line reflect the existence of two biochemical compartments in the muscle and differences in their buffering capacity. The observed splitting of the carnosine peak at 8 ppm supports the application of carnosine ^1^H MRS for the assessment of the pH separately in glycolytic and oxidative fiber‐containing muscle compartments.
